# *In situ* sol-gel synthesis of hyaluronan derivatives bio-nanocomposite hydrogels

**DOI:** 10.1093/rb/rbz029

**Published:** 2019-10-09

**Authors:** U D’Amora, A Ronca, M G Raucci, S M Dozio, H Lin, Y Fan, X Zhang, L Ambrosio

**Affiliations:** 1 Institute of Polymers, Composites and Biomaterials, National Research Council, Naples, Italy; 2 Institute of Science and Technology for Ceramics, National Research Council, Faenza, Italy; 3 School of Advanced Study “G. D’Annunzio”, University of Chieti-Pescara, Chieti Italy; 4 National Engineering Research Center for Biomaterials, Sichuan University, Chengdu, China

**Keywords:** hyaluronic acid derivatives, photocrosslinking, sol-gel, nanocomposites

## Abstract

The main driving idea of the present study was the comparison between two different chemical modifications of hyaluronic acid (HA) followed by the development of nanocomposite hydrogels directly *in situ* by biomineralization of photocrosslinkable HA polymers through sol-gel synthesis. In this way, it has been possible to overcome some limitations due to classical approaches based on the physical blending of inorganic fillers into polymer matrix. To this aim, methacrylated and maleated HA, synthesized with similar degree of substitution (DS) were compared in terms of mechanical and physico-chemical properties. The success of *in situ* biomineralization was highlighted by reflect Fourier transform infrared spectroscopy and thermogravimetric analysis. Furthermore, mechanical characterization demonstrated the reinforcing effect of inorganic fillers evidencing a strong correlation with DS. The swelling behavior resulted to be correlated with filler concentration. Finally, the cytotoxicity tests revealed the absence of toxic components and an increase of cell proliferation over culture time was observed, highlighting these bio-nanocomposite hyaluronan derivatives as biocompatible hydrogel with tunable properties.

## Introduction

Hyaluronic acid (HA), also named hyaluronan, is a linear polysaccharide consisting of alternating units of d-glucuronic acid and *N*-acetyl-d-glucosamine and it is found in all connective tissues of the body as one of the major components of the extracellular matrix (ECM) [1–3]. Due to its properties, HA is involved in many natural processes such as biological signaling, wound repair, morphogenesis and ECM organization [4, 5]. Other biological functions of HA include maintenance of the elastoviscosity of liquid connective tissues such as joint synovial and eye vitreous fluid, control of tissue hydration and water transport [6, 7]. However, HA results highly soluble at room temperature with high rate of degradation and low mechanical properties, depending on its molecular weight and location environment limiting its possible application as biomaterial for tissue engineering applications [8, 9]. Chemical modification of HA with subsequent crosslinking is one of the most used methods to improve the mechanical properties also reducing the degradation time *in vivo*. To chemically modify HA, the primary and secondary hydroxyl and the carboxylic acid functional groups have been targeted [4, 10]. Different techniques have been described widely in literature, offering a broad spectrum of options for the synthesis of hydrogels with various physico-chemical properties [11]. Among all the methods, esterification using alkyl succinic anhydrides or methacrylic anhydride (ME) is the most used techniques [12, 13]. The presence of methacrylate group enables the photocrosslinking of HA by free radical polymerization, obtaining hydrogels with longer degradation time and higher mechanical properties [14, 15]. HA and its derivatives have been clinically used for different medical applications such as ophthalmic surgical aid [16], in the treatment of knee osteoarthritis [3] and as scaffolds for bone and osteochondral repair [2, 17–20]. Traditionally, composite scaffolds have been obtained with different strategies involving physical mixing of inorganic micro-nanoparticles (hydroxyapatite, HAp) and natural or synthetic polymers [21, 22]. In this scenario, considering the limits of the traditional approaches (i.e. presence of inorganic aggregates in the polymer matrix), the *in situ* sol-gel process has been considered as a versatile new procedure that ensures a more controlled and finer distribution of crystallites in the polymer matrix [23]. Furthermore, the advantages of the *in situ* sol-gel synthesis include high purity of the final products, possibility to produce bioglasses [24], hybrid materials and bioceramics nanocrystals at low temperature and at different pH. By varying the pH conditions of the process, it is possible to produce HAp and other calcium phosphate (CaP) phases such as dicalcium phosphate anhydrous (DCP, CaHPO_4_ also known as monetite), a precursor of natural HAp in bone [23, 25]. In the present work, it has been assumed that the improved stability and mechanical properties of chemically modified HA sodium salt (HAs) in combination with biological features of CaP fillers, obtained by *in situ* sol-gel technique, can lead to a valuable biomaterial able to regenerate tissue interfaces. For this reason, two different chemical modifications of the HA, by methacrylic and maleic anhydride (MA), have been proposed to obtain photocrosslinkable hydrogels. Methacrylated (MEHA) and maleated hyaluronan (MAHA), with similar degree of substitution (DS), were synthesized, and compared in terms of physico-chemical, mechanical and biological behavior. Furthermore, bio-nanocomposite hyaluronan derivatives have been successfully synthesized by *in situ* sol-gel approach as biocompatible hydrogels.

## Materials and methods

### Chemical modification of HAs

**Figure 1:**
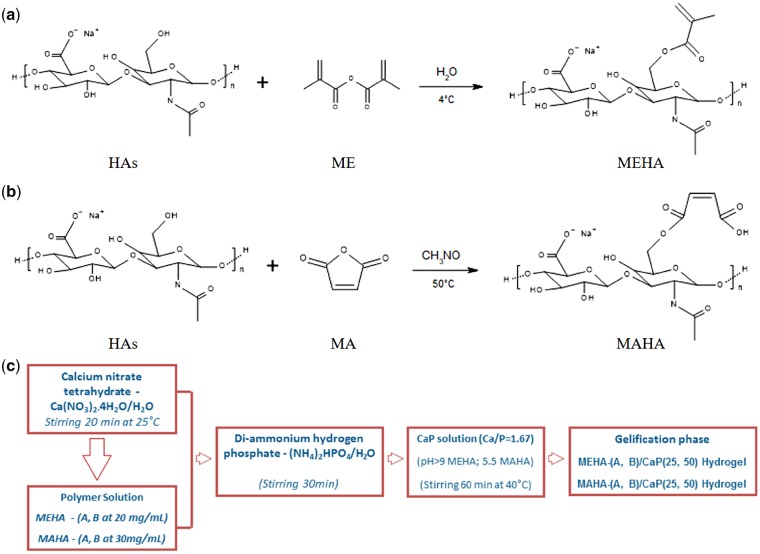
Reaction scheme of (a) MEHA, (b) MAHA) and (c) *in situ* sol-gel synthesis

HAs (M_w_=∼340 kDa from *Streptococcus equi*, Bloomage Freda Biopharm Co. Ltd., Shandong, China) was modified to graft photoactive polymerizable groups by reacting with ME (Sigma Aldrich) and MA (Kelong Chemical CO. Ltd). MEHA was synthesized following an adapted protocol from Bian *et al.* (Fig. 1a) [26]. HAs was dissolved in distilled water (dH_2_O) and stirred overnight at room temperature for complete dissolution and it was functionalized by reacting the primary hydroxyl groups (-OH) with methacrylic moieties at 4°C. The DS was varied by using an excess of 20 and 30 mol% ME per hydroxyl group, obtaining MEHA-A and MEHA-B, respectively. The pH of the solution was maintained between 8 and 9 by adding sodium hydroxide (NaOH, Sigma Aldrich) and the reaction was carried out overnight. MEHA solutions were precipitated into cold anhydrous ethyl alcohol (EtOH) and the supernatant was recovered by centrifugation and subsequent filtration. The isolated MEHA polymers were then washed three times with EtOH, dialyzed against pure water for 5 days and freeze-dried. Similarly, MAHA was obtained by reacting primary -OH groups with MA (Fig. 1b). HAs was dissolved at a concentration of 1% (wt/v) in dry formamide (CH_3_NO, Sigma Aldrich) at 40°C under a nitrogen atmosphere (N_2_), and stirred until fully dissolved. MA was dissolved in dry formamide, then it was added to the HAs solution and the reaction was carried out for 24 h at 40°C in N_2_. The DS was modified by varying the amount of MA existent in the reaction mixture (excess of 15 and 20 mol% to the primary -OH groups), obtaining MAHA-A and MAHA-B, respectively. MAHA polymers were then precipitated into EtOH, washed and freeze-dried following the same step described above.

### Biomineralization by *in situ* sol-gel synthesis

Composite materials with a polymer to filler weight ratio (wt/wt) 75/25 (CaP25) and 50/50 (CaP50) were produced by *in situ* sol-gel synthesis. Calcium nitrate tetrahydrate Ca(NO_3_)_2_ × 4H_2_O (Jin Shan Hua Shi, China) and di-ammonium hydrogen phosphate (NH_4_)_2_HPO_4_ (Jin Shan Hua Shi, China) were used as precursors of CaP and dH_2_O as solvent (Fig. 1c). In particular, MEHA and MAHA, at different DS, used as polymer matrix, were dissolved in dH_2_O at the concentration of 20 and 30 mg/mL, respectively, and a solution of Ca(NO_3_)_2_ × 4H_2_O (Ca solution; 3.00 M) was drop-wise added to the polymer solution at 40°C. After that, di-ammonium hydrogen phosphate (P solution, 3.58 M), previously dissolved in dH_2_O, was added to the polymer solution in order to achieve a Ca/P ratio of 1.67 (mol/mol) similar to stoichiometric HAp. After 30 min, the medium alkalinity was adjusted by the addition of ammonium hydroxide (NH_4_OH, Sigma Aldrich) up to pH 9 for MEHA, while for MAHA-based composite hydrogels, the pH value was maintained at pH = 5.5. MEHA-(A, B)/CaP(25, 50) and MAHA-(A, B)/CaP(25, 50) solutions were placed in a shaking incubator at 100 rpm, 37°C until gelling occurred.

### Hydrogel preparation

Freeze-dried MEHA and MAHA, at different DS, were dissolved in dH_2_O containing 0.1% (wt/v) 2-hydroxy-4′-(2-hydroxyethoxy)-2-methylpropiophenone (Irgacure 2959, Sigma Aldrich) used as a biocompatible photoinitiator. Solutions at 2 and 3% (wt/v) of MEHA and MAHA, respectively, were used. To obtain disc-shaped samples, MEHA and MAHA solutions (150 μL) were transferred to a Teflon mold between two glass plates [diameter (d) = 6.0 ± 0.1; height (h) = 2.0 ± 0.2 mm] and then irradiated with ultraviolet (UV) light [OmniCure S1500 (USA), λ: 365 nm, ∼16 mW/cm^2^] for 60 and 120 s, respectively. Finally, cross-linked samples were detached from the mold and stored at 4°C in reverse osmosis water before testing. Composite samples were obtained in a similar way. Briefly, Irgacure 2959 was added into the composite gel solution, as prepared in Section ‘Biomineralization by *in situ* sol-gel synthesis’, at a concentration of 0.1% (wt/v). About 200 µL of the composite gel solution was transferred into the cylindrical mold and the exposure time was increased to 180 s due to the shield effect of CaP fillers. After exposure, the samples were stored at 4°C.

### Physico-chemical analysis

#### Attenuated total reflect Fourier transform infrared spectroscopy

Attenuated total reflect Fourier transform infrared (ATR FT-IR) spectroscopy (ThermoFisher Nicolet IS10, USA) was employed to identify the functional groups attached to HAs and the presence of the inorganic fillers. Dried composite materials were scanned from 600 to 2000 cm^−1^ with a resolution of 2 cm^−1^. The neat HAs powder was also scanned under the same condition and served as control.

#### Thermogravimetric analysis

Thermogravimetric analysis (TGA) was performed to evaluate the thermal stability and the effective weight percentage of inorganic content in the composite materials using a TA Instruments TGA model 2950. Dried specimens (4–7 mg) were heated under N_2_ flow from room temperature to 700°C at a heating rate of 10°C/min. In particular, the residuals found $(W_{\mathrm{Ci}}$) in the TGA thermograms were compared with the theoretical amounts$(W_{\mathrm{TCi}}$) of *in situ* synthesized CaP, calculated by using the following equation:

$$W_{\mathrm{TCi}}=W_{\mathrm{Ci}}-\left(\frac{i*W_{N}}{100}\right)$$

with

*i* = 75 or *i* = 50 for MEHA- or MAHA-based composites with a polymer to filler weight ratio (wt/wt) 75/25 (CaP25) or 50/50 (CaP50), respectively; $W_{N}$ is the residual found for MEHA or MAHA.

#### Swelling studies

Completely dried hydrogel neat and nanocomposite samples were weighted (w_0_) and left to swell in physiological condition up to 3 days (pH ≈ 7.4, T ≈ 37°C). The swollen hydrogels were then taken out from phosphate-buffered saline (PBS) at fixed time, the surface adsorbed water was removed by filter paper, the weight was recorded (w_t_) and the samples placed in PBS again. The swelling ratio (Q), expressed as mean ± standard deviation (SD, *n* = 3), was obtained using the following expression.

(1) $$Q=\left(\frac{w_{t}-w_{0}}{w_{0}}\right)$$

### Morphological analysis

#### Scanning electron microscopy

Neat and composite hydrogels were observed by SEM (S-800; Hitachi). Before observation, hydrogels were washed with MilliQ water, frozen in liquid nitrogen, lyophilized in a vacuum freeze drier (VirTis) for 48 h and cut. The lyophilized samples were coated with an ultrathin layer of Au/Pt by using an ion sputter and then observed by SEM. SEM-energy dispersive X-ray spectroscopy (EDS) mapping was also used to assess the dispersion of the CaP fillers in the polymer matrix.

#### Transmission electron microscopy

Transmission electron microscopy (TEM) images were collected using a Hitachi H-9000NAR model instrument operated at an accelerating voltage of 100 kV. Samples were prepared by placing a drop of the composite gel suspensions (diluted in dH_2_O and dispersed by ultrasonic waves) onto carbon coated copper grids, dried in air and loaded into the electron microscope chamber. After collecting the images, length and width of CaP fillers were measured using commercial imaging software (ImageJ, National Institutes of Health, Bethesda, MD).

### Mechanical analysis

#### Dynamic mechanical analysis

The dynamic mechanical analysis (DMA) was carried out using a NETZSCH DMA 242 C instrument in compression mode at a frequency of 1 Hz simulating the normal physiological stride frequency. The tests were performed at room temperature in air atmosphere. A strain amplitude of 50 μm, a preload force of 0.001 N and a force track of 120% were used. The testing procedure only required a few minutes and dehydration over such a short-time scale was not anticipated. Five cylindrical samples (d = 6.0 ± 0.1; h = 2.0 ± 0.2 mm) were used to characterize neat and composite hydrogels, at different DS and inorganic filler concentration. The storage modulus (E′) and loss modulus (E″) of each sample were reported as mean value ± SD.

#### Compression test

Unconfined compression tests were carried out on cylindrical structures (d = 12.0 ± 0.2 mm; h = 10.0 ± 0.3 mm) in the wet state and at room temperature. Neat and composite hydrogels, at different DS and filler concentration, were compressed by the upper plate connected to a load cell at 1 mm/min up to a strain level of 0.5 mm/mm. The initial compressive modulus (E) was determined by the average slope in a range of 0–10% strain from the stress/strain curve. The maximum stress (σ_max_) was determined from the peak of the stress/strain curve. All the tests were performed on five cylindrical specimens for every composition, using an INSTRON 5566 testing machine (Bucks, UK).

### Biological investigation

#### In vitro *cell culture*

Biological assays were performed using murine fibroblast (L929) cell lines. L929 were cultured in plastic culture dishes in Dulbecco’s Modified Eagle’s Medium (DMEM)-high glucose supplemented with 10% fetal bovine serum (FBS), antibiotic solution (streptomycin 100 µg/mL and penicillin 100 U/mL, Sigma Aldrich) and 2 mM l-glutamine. The cells were incubated at 37°C in a humidified atmosphere with 5% CO_2_ and 95% air humidity. Hydrogel-based materials (d = 6.0 ± 0.3 mm; h = 3.0 ± 0.2 mm) were sterilized by ethanol 70% and UV irradiation (1 h).

#### Indirect cytotoxicity assay

Indirect test was performed by adding 2.5 ml of DMEM solution to each 0.1 g of material according to the ISO 10993-5 guidelines. The materials were placed in a shaking incubator (100 rpm) for a period of 24 h (elution time). The eluents were removed and 100 µL pipetted into sterile 96-well cell culture plates (Falcon, USA) previously seeded with L929 cells (80% of confluence) and incubated for an exposure time of 1 and 7 days at 37°C, 5% CO_2_ and 95% air humidity. The cell vitality was determined using an Alamar blue assay (AbD Serotec, Milano, Italy) based on the metabolic activity of live cells. The absorbance was measured at 570 and 600 nm by spectrophotometer (Victor X3, Perkin Elmer) at 1 and 7 days after cell seeding.

#### Direct cytotoxicity assay

To evaluate the direct effect of material surface on cell vitality and proliferation, materials were pre-soaked for 24 h in culture medium in order to equilibrate the pH to physiological value and then, each sample was seeded with a 20 μL drop of cell suspension (5 × 10^4^ cells/sample). The medium in cell-load materials culture plates was removed after culturing for 1 and 3 days and *in vitro* cell vitality was evaluated with CCk-8 assay, according to the manufacturer’s protocol. Finally, absorbance was measured at 450 nm by a spectrophotometer (ThermoScientific).

### Statistical analysis

Results were expressed as mean ± SD and plotted. Statistical analyses were performed by the GraphPad Prism software (version 7.0). Statistical significances between the time points were calculated using the two-way analysis of variance (ANOVA) test.

## Results and discussions

### Physico-chemical analysis

**Figure 2:**
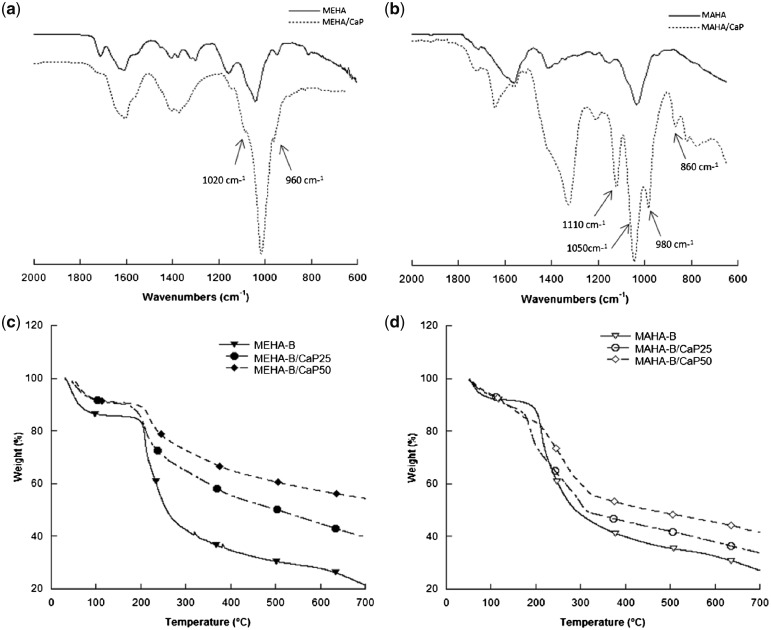
ATR FT-IR Spectra of (a) MEHA- and (b) MAHA composite hydrogels and TGA results of (c) MEHA-B and (d) MAHA-B

^1^H NMR spectra of MEHA and MAHA, reported in Supplementary Fig. S1, showed the characteristic peaks of methacrylic and maleic moieties, respectively. By varying the amount of methacrylic and MA used in the reaction, the results demonstrated that DS could be successfully modified. As reported in Supplementary Table S1, the present study suggested that 51.13 ± 4.6% and 79.96 ± 2.49% of functionalization were achieved under the reaction conditions of 20 and 30 molar excess of ME and reaction time of 24 h. Similarly, for MAHA, DS of 54.14 ± 2.62% and 85.49 ± 4.86% were achieved by using 15 and 20 molar excess of MA and reaction time of 24 h (Table 1). Results highlighted that by finely tuning the MA/HAs and ME/HAs ratios, it was possible to obtain MEHA and MAHA, respectively, with a DS in the same range. Furthermore, ATR FT-IR performed on neat synthesized polymers (Supplementary Fig. S2) confirmed that the functional moieties are successfully branched on the polysaccharide chain and the DS was influenced by the reaction parameters. ATR-FTIR analysis was also performed to evaluate the chemical composition of composite materials (Fig. 2a and b). In the spectrum, the main chemical groups of HAp, ${\text{PO}}_{4}^{3}$^−^, OH^−^, ${\text{CO}}_{3}^{2}$^−^ are present, as well as ${\text{HPO}}_{4}^{2}$^−^ that characterizes the presence of DCP. In particular, in Fig. 2a, the peak at 1020 cm^−1^ and the shoulder at 960 cm^−1^ are typical of asymmetrical stretching (*v_3_*) and bending (*v_1_*) modes of ${\text{PO}}_{4}^{3}$^−^. The OH^−^ and ${\text{CO}}_{3}^{2}$^−^ are covered by the typical peaks of MEHA (Supplementary Fig. S2). Meanwhile, in Fig. 2b, the sharp ${\text{PO}}_{4}^{3}$^−^ peaks developed at 1110, 1050, 980 and 860 cm^−1^ demonstrated the DCP formation. TGA of chemically modified HAs, and composites with different composition was carried out at temperatures ranging from 20 to 700°C under N_2_. Fig. 2c-d show thermogravimetric spectra of MEHA-B- and MAHA-B-based hydrogels. The first weight loss, between 50°C and 150°C, can be ascribed to residual water trapped into the hydrogel due to the hydroxyl groups of the polymer chains. The second step, between 250°C and 350°C, is related to the thermal degradation of the backbone chains of the polymer matrix. Taking into consideration that the CaP phase is stable at 700°C [27], the quantification of residual masses allows to assess the precise composition of the composites. For neat polymers, the remaining amount found at 700°C can be probably related to the formation of coke residue during thermal polymer degradation as demonstrated in previous works [28, 29]. The residual amount in the networks at 700°C was, respectively, 21.5 wt% for MEHA-B, 39.7 wt% for MEHA-B/CaP25 and 54.4 wt% for MEHA-B/CaP50 (Fig. 2c). With regard to MAHA, it was 27.0 wt% for MAHA-B, 33.8 wt% for MAHA-B/CaP25 and 41.6 wt% for MAHA-B/CaP50 (Fig. 2d). The residual found in the TGA thermograms for MEHA composites is in agreement with the theoretical amount of *in situ* synthesized CaP (23.6 and 43.7 wt% for MEHA-B/CaP25 and MEHA-B/CaP50, respectively), while for MAHA-based composites, this value is lower (13.6 and 28.1 wt% for MAHA-B/CaP25 and MAHA-B/CaP50, respectively).

**Table 1:** Reaction parameters and DS of MEHA and MAHA as calculated by ^1^H NMR

Samples	HAs/H_2_O (wt/v)	HAs/CH_3_NO (wt/v)	ME/HAs (mol/mol)	MA/HAs (mol/mol)	Temperature/atmosphere	pH	**DS** ^a^ **(%)**
**MEHA-A**	1/100		20/1		4°C/Air	8.0–9.0	51.13 ± 4.61
**MEHA-B**	1/100		30/1		4°C/Air	8.0–9.0	79.96 ± 2.49
**MAHA-A**		1/100		15/1	40°C/N_2_		54.14 ± 2.62
**MAHA-B**		1/100		20/1	40°C/N_2_		85.49 ± 4.86

^a^DS have been reported as mean value ± SD, *n* = 3.

To investigate the effect of DS and CaP amount on the swelling behavior of the hydrogels, the swelling ratio of the neat and composite samples was assessed.

**Figure 3:**
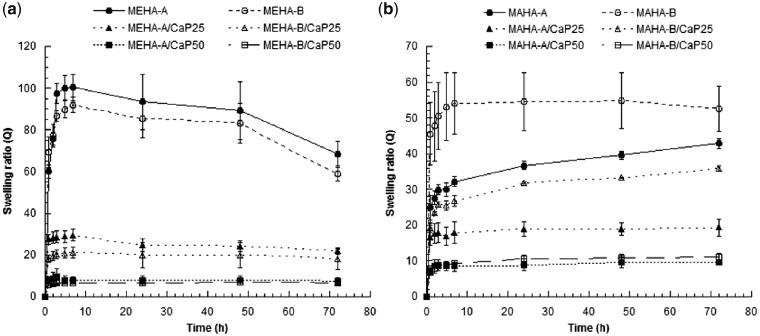
Swelling behavior for (a) MEHA and (b) MAHA

Figure 3 shows the results from swelling test in PBS at physiological conditions after 3 days. Both hydrogels, independent of DS and inorganic phase concentration, were almost stable in the first three days, except for neat MEHA hydrogels that showed a slight reduction of swelling ratio after 2 days. This behavior could be ascribed to an initial degradation of MEHA. This effect was not evident for the composite samples because the HAp nanoparticles stabilize the structure of the hydrogel reducing the water absorption. In fact, for MEHA-based composites (Fig. 3a), the swelling degree was found to increase at short time (7–8 h), following a plateau region till 3 days. With regard to DS, no statistical differences were found between neat and both composites, respectively, with higher and lower DS (A, B). Meanwhile for MAHA, a different behavior was observed. From Fig. 3b, it is clear that for neat hydrogel, the swelling ratio increased at each time point by increasing the DS (*P* < 0.05), while at 3 days no statistical differences were observed. For MAHA-(A, B)/CaP25 composite hydrogels, Q_MAHA-A_ was statistically lower than Q_MAHA-B_ (*P* < 0.001) at each time point, while for MAHA (A, B)/CaP50, no statistical differences were observed. In general, it can be assumed that the highly hydrophilic carboxyl groups (COOH), belonging to MA grafted on the polymer chain, yield an enhanced water absorption capacity inside the network. This means that the negative effect due to the crosslinking on the swelling capacity should be balanced by the positive effect related to the hydrophilicity of MA groups. The composite hydrogels showed significantly decreased swelling ratio compared with neat materials (*P* < 0.001). Figure 3 shows that by increasing the amount of CaP within the hydrogel, the swelling ratio decreased. With increasing CaP concentration, the relative content of polymer decreases, which is responsible for the decreased swelling ratio. Moreover, swelling of the hydrogels involves large-scale segmental motion, and the *in situ* synthesized CaP could contract and restrict the chain mobility contributing to the decreased swelling ratio. Neat MEHA hydrogels achieved a maximum of swelling ratio which was almost double than MAHA ones.

### Morphological analysis

**Figure 4:**
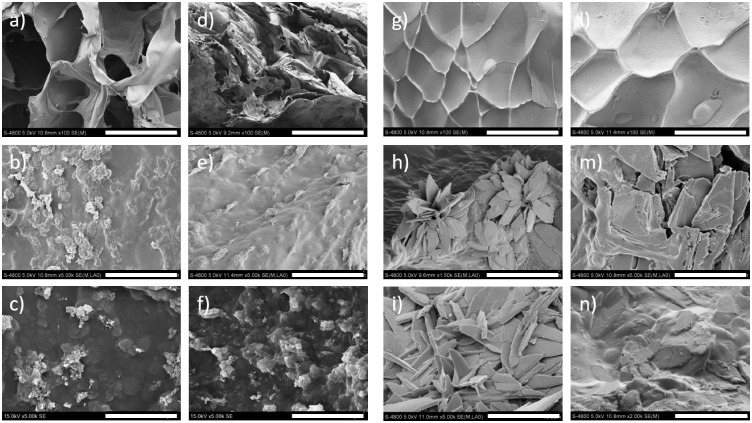
SEM Analysis of: (a) MEHA-A, (b) MEHA-A/CaP25, (c) MEHA-A/CaP50, (d) MEHA-B, (e) MEHA-B/CaP25, (f) MEHA-B/CaP50, (g) MAHA-A, (h) MAHA-A/CaP25, (i) MAHA-A/CaP50, (l) MAHA-B, (m) MAHA-B/CaP25, (n) MAHA-B/CaP50. Scale bars: (a, d, g, l) 500 µm; all the others 10 µm

Detailed microstructural studies using SEM and TEM gave further insight into the shape and size of the formed phases. SEM analyses were carried out on the surface and on the cross-section of the samples. The CaP fillers were homogenously distributed in all samples, as also highlighted by the EDS mapping and TB/AR staining images (Supplementary Figs S3 and S4,), with no evidence of microscale clusters. This can be ascribed to the *in situ* chemical synthesis that ensures a finer distribution of the inorganic fillers. SEM investigations performed on MEHA-based composites (Fig. 4a–f) showed the typical shape of HAp, independent of DS. Moreover, the presence of microporosity due to solvent evaporation and ammonium nitrate removal was also observed.

**Figure 5:**
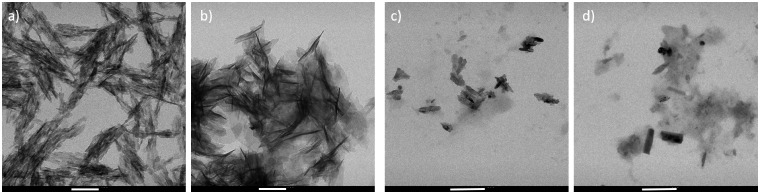
TEM analysis of: (a) MEHA-B/CaP25, (b) MEHA-B/CaP 50, (c) MAHA-B/CaP25, (d) MAHA-B/CaP50. Scale bars: (a, b) 100 nm; (c, d) 200 nm

On the other side, MAHA-based samples were produced maintaining the pH at 5.5. Interestingly, results showed a different morphology of the inorganic fillers (Fig. 4g–n). They highlighted a plate-like, typical of DCP (brushite and monetite), which is recognized to be a stable phase under more acidic conditions. Because of its good biocompatibility and bioactivity, monetite is usually used as resorbable bone replacement materials [30, 31], and it is one important component of CaP cements. Furthermore, the monetite has also been considered for its relatively more rapid resorption rate *in vivo*. TEM analyses were performed in order to obtain an image strongly magnified of the gel suspension and quantify the shape and the size of the crystals. As representative images, Fig. 5 reports TEM analyses performed on MEHA-B and MAHA-B composites. MEHA-B/CaP25 composites showed a length of about 86.9 ± 5.9 nm and a thickness of 5.1 ± 1.8 nm. By increasing the filler concentration, the length and thickness increased to 171.0 ± 33.9 nm and 6.6 ± 2.0 nm, respectively (Fig. 5a and b). This can be related to a more packed structure that can lead to an agglomerate effect of the inorganic phase. Similarly, for MAHA-based composites, inorganic fillers showed a size slightly smaller than MEHA-based ones; even if the same trend was observed. By increasing the CaP concentration, the inorganic filler size increased from 56.6 ± 8.3 nm (length) and 22.6 ± 4.7 nm (width) to 126.2 ± 24.0 nm (length) and 30.9 ± 8.9 nm (width) (Fig. 5c and d).

### Mechanical analysis

**Figure 6:**
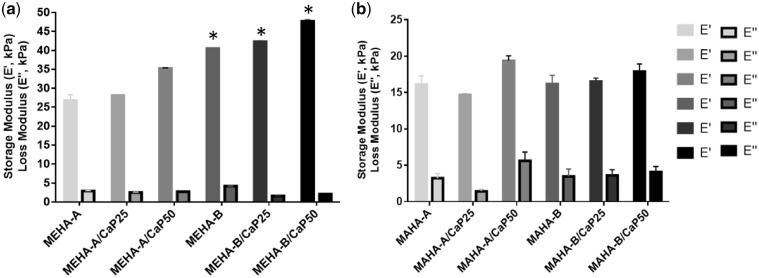
DMA results of: (a) MEHA and (b) MAHA disc-shaped samples, (*n* = 5). *P < 0.01 by comparing the respective materials with higher and lower DS

Results from DMA performed on MEHA and MAHA and their composites are reported in Fig. 6. Mechanical properties of MEHA seem to be strongly influenced by the DS. In particular, all the MEHA-B-based composites showed values of the storage modulus generally higher than the respective MEHA-A-based ones (*P* < 0.01). Indeed, E′ spans from 26.7 kPa for MEHA-A to 40.1 kPa for MEHA-B. The presence of CaP improved the mechanical properties. In particular, the storage modulus increased by increasing the particle concentration. Taking MEHA-B as an example material group, E′ spans from 42.3 kPa (CaP25) to 47.7 kPa for MEHA (CaP50) (Fig. 6a). Instead, the mechanical behavior of MAHA hydrogels seems to be more dependent to the CaP concentration than to the DS. Indeed, by increasing the CaP amount, the mechanical properties increased and the samples showed a value of E′ of 19.3 kPa and 20.9 kPa for MAHA-A and MAHA-B, respectively (Fig. 6b). As expected, for all the materials, the loss moduli are lower than their storage moduli, suggesting their gel-like behavior. Furthermore, by comparing MEHA and MAHA, MEHA-B samples highlighted a storage modulus generally higher than MAHA (*P* < 0.01).

Compression tests were performed on MEHA- and MAHA- (A and B), as well as on their composites (CaP25 and CaP50) hydrogels. Results are reported in Table 2 and show that for all the synthesized MEHA-based materials, mechanical properties, in terms E increased by increasing DS (*P* < 0.01) and CaP concentration (*P* < 0.01), while for σ_max_, a statistical difference was only observed by varying the DS (*P* < 0.001). With regard to MAHA-based hydrogels, a statistical difference was observed for E by varying the CaP amount (*P* < 0.01), while for σ_max_, a statistical difference was assessed between neat and composite hydrogels (*P* < 0.001). As an example, for MEHA, by increasing the DS, E and σ_max_ increased from 23.3 kPa to 29.6 kPa and from 7.9 kPa to 14.5 kPa, respectively. Similarly, the maximum strain (ε_max_) slightly increased from 0.22 mm/mm to 0.34 mm/mm. Regardless of the DS achieved, the inorganic fillers clearly acted as reinforcement for the polymer matrix in a concentration dependent way, as evidenced by the increased values of E and σ_max_. The introduction of CaP caused discontinuities at the filler/matrix interface; this effect is due to the difference in the ductility between the polymeric matrix and purely inorganic phase. Therefore, the different ductility of the neat and composite hydrogels should be responsible for the distinct mechanical behavior during compression, as shown by the increased value of ε_max_ (*P* < 0.01). A similar trend was also evidenced by MAHA hydrogels. However, MAHA materials showed a significant lower compressive modulus (*P* < 0.001) if compared with the respective MEHA hydrogels.

**Table 2:** Results obtained from compression tests performed on MEHA and MAHA, at different DS (A and B) and their composite hydrogels (CaP25 and CaP50)

Materials	E (kPa)	σ_max_ (kPa)	ε_max_ (mm/mm)
**MEHA-A**	23.3 ± 2.1	7.9 ± 1.8	0.22 ± 0.03
**MEHA-A/CaP25**	27.5 ± 1.7	9.5 ± 1.4	0.25 ± 0.04
**MEHA-A/CaP50**	30.3 ± 3.1	11.5 ± 2.3	0.26 ± 0.07
**MEHA-B**	29.6 ± 3.5	14.5 ± 3.6	0.34 ± 0.03
**MEHA-B/CaP25**	32.1 ± 1.4	15.9 ± 2.3	0.39 ± 0.02
**MEHA-B/CaP50**	36.9 ± 3.1	20.9 ± 3.2	0.45 ± 0.05
**MAHA-A**	10.1 ± 2.1	3.3 ± 0.9	0.32 ± 0.04
**MAHA-A/CaP25**	16.1 ±2.5	29.8 ± 8.2	0.45 ± 0.07
**MAHA-A/CaP50**	22.1 ± 3.3	32.4 ± 7.6	0.47 ± 0.05
**MAHA-B**	12.7 ± 3.9	3.9 ± 0.9	0.31 ± 0.06
**MAHA-B/CaP25**	19.3 ±3.5	36.1 ± 5.4	0.46 ± 0.07
**MAHA-B/CaP50**	23.1 ± 4.5	40.0 ± 3.2	0.47 ± 0.05

Compressive modulus (E), maximum stress (σ_max_) and maximum strain (ε_max_) are reported as mean value ± SD, *n* = 5

### Biological investigation: indirect and direct cytotoxicity

**Figure 7:**
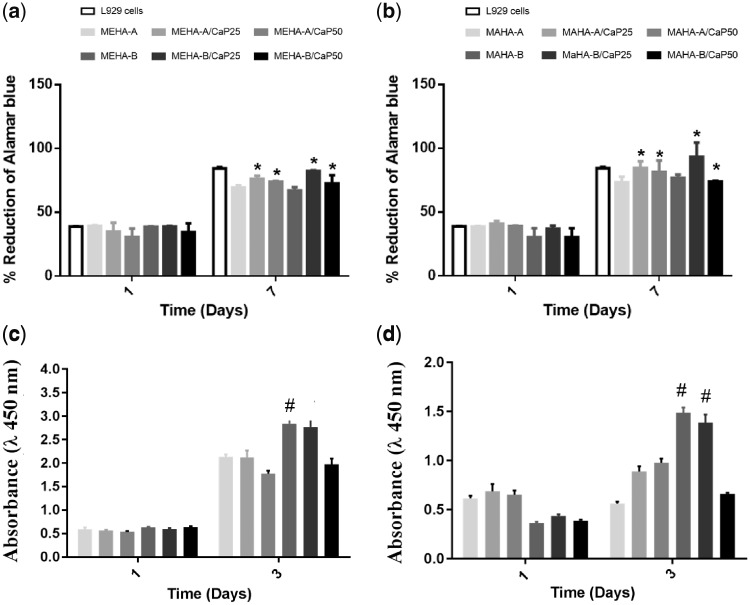
Indirect quantitative by alamar blue assay results on: (a) MEHA- and (b) MAHA-based hydrogels (at 1, 7 days, *n* = 4). 2 D plate surface was used as control. *P < 0.01 versus the respective neat polymer substrate at each time point. Direct quantitative by CCK-8 assay results on: (c) MEHA- and (d) MAHA-based hydrogels (at 1 and 3 days, *n* = 4). #*P* < 0.05 versus all the samples at the same day. Data are expressed as mean ± SD of four experiments.

The biocompatibility of the hydrogel materials was determined quantitatively using methods, which assessed cell metabolic function and proliferation such as Alamar Blue and CCK-8 assays. The results of indirect test of L929 cells and eluents collected after 24 h, demonstrated that MEHA- and MAHA-based hydrogels did not release any toxic components that could have a negative effect on cell vitality. Indeed, no significant differences were observed between the cells expanded in conditioned medium and control at 1 and 7 days (Fig. 7a and b). On the other hand, the results from direct contact with material surface demonstrated an increasing of cell proliferation over culture time with the highest value for MEHA-B and MEHA-B/CaP25 (Fig. 7c). Similarly, for MAHA-based hydrogels, the best behavior was obtained at day 3 for MAHA-B and MAHA-B/CaP25 (Fig. 7d).

**Figure 8:**
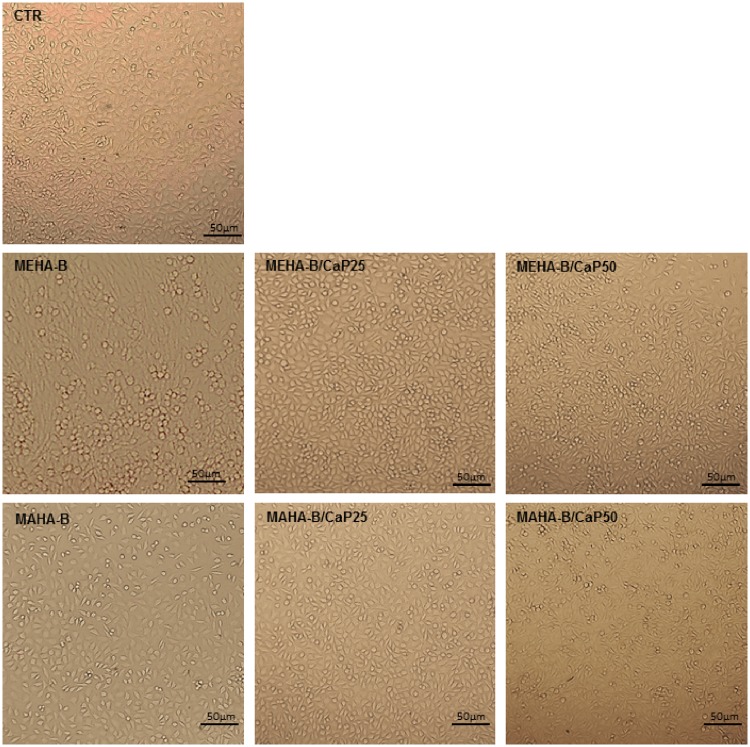
Light microscope images (×10) of cells growth in conditioned media derived from MEHA-B, MAHA-B hydrogels and their composites. Scale bar: 50 μm

The qualitative images acquired by light microscope (Fig. 8) highlighted the good vitality of cells incubated in conditioned media, obtained from neat and composite hydrogels, thus confirming the indirect cytotoxicity results.

## Conclusions

Nanocomposite hydrogels, based on chemically modified HAs and *in situ* sol-gel CaP, were successfully developed and characterized in terms of physico-chemical, morphological, mechanical and biological properties. To this aim, HAs were first chemically functionalized with photocrosslinkable moieties by reacting with methacrylic and MA to obtain a MEHA and MAHA with similar DS. Composite hydrogels with different polymer to inorganic filler weight ratios were synthesized at room temperature by *in situ* sol-gel synthesis. ATR-FT IR analysis showed the success of chemical reaction, evidencing for MEHA the presence of HAp as well as DCP. Meanwhile, for MAHA, the analyses demonstrated only DCP formation. The results were also confirmed by morphological analyses. Indeed, needle like nano-HAp was mostly found for MEHA, while typical plate-like monetite for MAHA. SEM analysis showed a good distribution of nanometer particles within the hydrogel, without the presence of microscale clusters. Mechanical properties were strongly correlated both with DS and inorganic filler amount, while DS-dependent swelling behavior was observed only for MAHA and MAHA-based composite with the lowest CaP concentration. On the contrary, for both hydrogels, an increased CaP concentration within the hydrogel led to a decreased swelling ratio. The biocompatibility of the hydrogels was quantitatively assessed by indirect and direct methods, which assessed cell metabolic function and proliferation. The results demonstrated that MEHA- and MAHA-based hydrogels did not release any toxic components, and an increase of cell proliferation over culture time was observed. In particular, the highest proliferation value was obtained for HA derivatives characterized by the highest DS and the lowest CaP amount. The overall results highlighted the possibility to tune the DS of the polymer matrix as well as the inorganic phase concentration of the polymer-based materials, obtaining bio-nanocomposite hydrogels characterized by a wide range of properties.

## Supplementary Material

rbz029_Supplementary_Data
