# Supplementary material for: In situ sol-gel synthesis of hyaluronan derivatives bio-nanocomposite hydrogels
Source: Regen Biomater. 2019 Oct 9;6(5):249–58. doi: 10.1093/rb/rbz029 (PMC6783703; doi:10.1093/rb/rbz029)
Supplement: rbz029_Supplementary_Data [file rbz029_supplementary_data.docx]

**Supplementary Information**

**In situ sol-gel synthesis of hyaluronan derivatives bio-nanocomposite hydrogels**

**U. D’Amora^1*^, A. Ronca^1*^, M.G. Raucci^1^, S. M. Dozio^2,3^, H. Lin^4^, Y. Fan^4^, X. Zhang^4^, L. Ambrosio^1^**

^1^ Institute of Polymers, Composites and Biomaterials, National Research Council, Naples Italy

^2^ Institute of Science and Technology for Ceramics, National Research Council, Faenza, Italy

^3^ School of advanced study “G. D’Annunzio”, University of Chieti-Pescara, Chieti Italy

^4^ National Engineering Research Center for Biomaterials, Sichuan University, Chengdu, China

**^*^**These authors contributed equally to this work

***Corresponding Author:**

Ugo D’Amora; Alfredo Ronca;

E-mail: [ugo.damora@cnr.it](mailto:ugo.damora@cnr.it); [alfredo.ronca@cnr.it](mailto:alfredo.ronca@cnr.it)

Phone: +390812425939

Fax: +390812425932

**Materials and Methods**

**Physico-chemical analysis**

*^1^H Nuclear magnetic resonance of modified HAs (NMR)*

^1^H NMR was used to determine the DS of the modified HAs. The HAs derivatives (both MEHA and MAHA, with different DS) were dissolved in deuterium oxide (D_2_O) with a concentration of 5-6 mg/mL by vigorously shaking at 7000 rpm for 20 min, and transferred into NMR tubes. The spectra were recorded at a frequency of 400 MHz using a Bruker AVIII 400HD nuclear magnetic resonance spectrometer (Swiss). Phase and baseline corrections were applied before obtaining the areas (integrals) of purely absorptive peaks.

**Morphological investigation**

*Toluidine Blue (TB) and Alizarin Red (AR) Staining of composite hydrogels*

In order to visualize hyaluronic acid and CaP distribution, composite hydrogels were stained with Toluidine Blue and Alizarin Red. In particular, the structures were kept for 3 days in paraformaldehyde 4% (wt/v) (Sigma Aldrich). The samples were gradually dried in ethanol/distilled water solution at different ratio (70/30, 80/20, 90/10, 100/0) for 30 minutes each. After that, they were treated with xylene (Sigma Aldrich) for 1h. Samples were embedded in paraffin, stored at 4 °C overnight and then they were cut in slices with a thickness of 5 μm each. Slices were washed two times in xylene for 15 min and then they were re-hydrated in ethanol/water solution at different ratios (100/0, 90/10, 80/20, 70/30) for 5 minutes each. Finally, they were washed two times in dH_2_O for 5 min. Slices were stained with TB 1% (wt/v) (Sigma Aldrich) for 5 min, and AR 0.2% (wt/v) (Sigma Aldrich) for 2 min. After washing, they were fixed with resin and observed with the optical microscope.

**Results and Discussion**

**Physico-chemical analysis**

The DS of the modified HAs (MEHA and MAHA) were determined by ^1^H NMR. The spectra (Fig. S1) showed characteristic peaks of HAs at 1.9 ppm attributed to the proton of the methyl (-CH_3_) group, while the peaks in the region of 3.3–5.6 ppm correspond to the protons from the D-glucuronic and N-acetyl glucosamine units (1).

Table S1: Peak area and DS as calculated by ^1^H NMR

| **Samples** |  | **Area [1]** | **Area [2]** | **Area [2+2’]** | **DS***  (%) | **Mean ± SD**  (%) |
| --- | --- | --- | --- | --- | --- | --- |
| **MEHA-A** | 1 | 1 | 5.33 | 10.33 | 56.27 | 51.13±4.61 |
|  | 2 | 1 | 6.34 | 11.34 | 47.35 |  |
|  | 3 | 1 | 6.03 | 11.03 | 49.78 |  |
| **MEHA-B** | 1 | 1 | 3.88 | 8.88 | 77.34 | 79.96±2.49 |
|  | 2 | 1 | 3.74 | 8.74 | 80.25 |  |
|  | 3 | 1 | 3.65 | 8.65 | 82.30 |  |
| **MAHA-A** | 1 | 1 | 5.7 | - | 52.36 | 54.14±2.62 |
|  | 2 | 1 | 5.2 | - | 57.14 |  |
|  | 3 | 1 | 5.6 | - | 52.91 |  |
| **MAHA-B** | 1 | 1 | 3.32 | - | 90.36 | 85.49±4.86 |
|  | 2 | 1 | 3.51 | - | 85.47 |  |
|  | 3 | 1 | 3.72 | - | 80.65 |  |

For MEHA, two peaks were evidenced, at approximately 5.6 and 6.1 ppm, which correspond to the introduced methacrylate moieties. Figure S1 also showed the ^1^H NMR spectra of MAHA with characteristic peaks at 5.9 and 6.6 ppm related to the vinylidene proton of the grafted maleic moiety. The DS of MEHA and MAHA were calculated by comparing the integral area of protons of unsaturated bonds (methacrylated and maleated moieties) with methyl (-CH_3_) peak. The peak area and relative DS expressed as mean±SD are reported in Table S1.


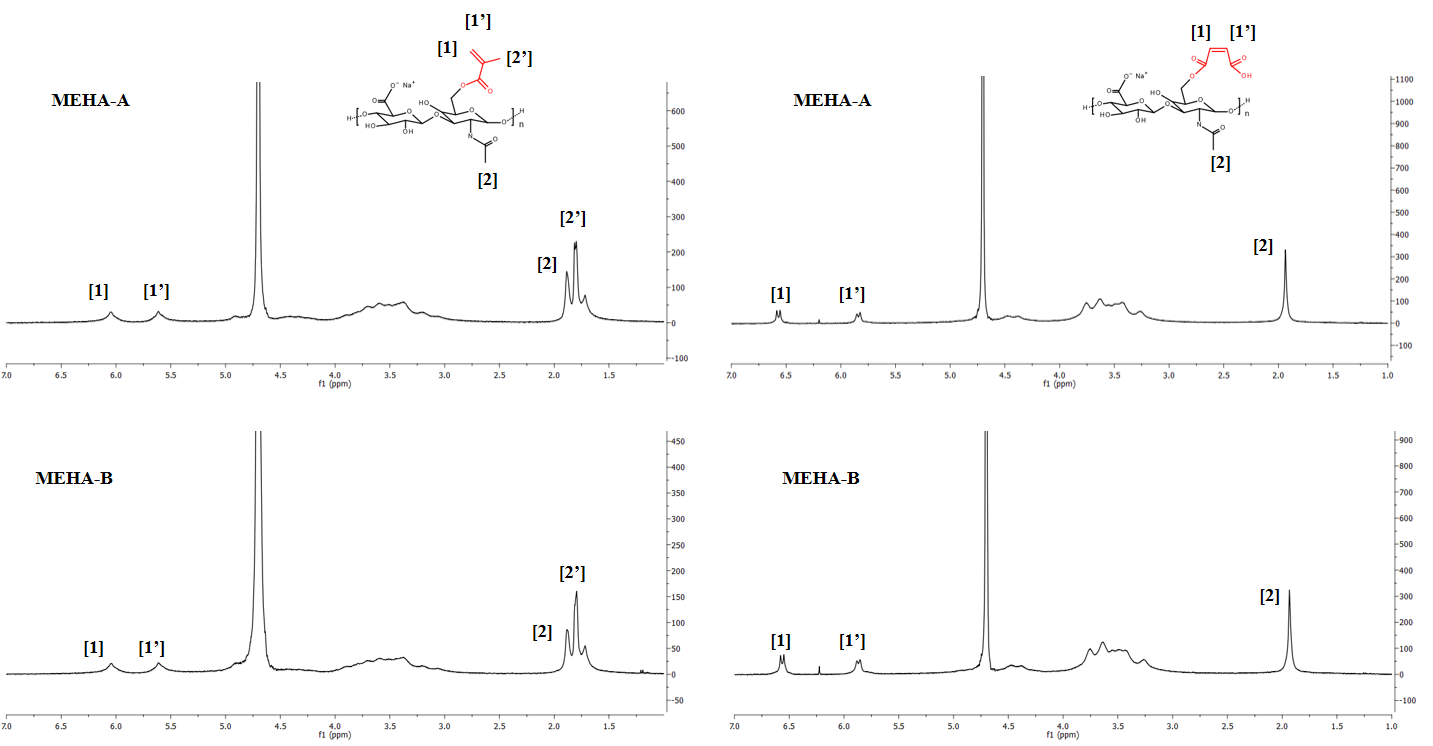


Figure S1. Representative ^1^H NMR spectra of MEHA and MAHA at different DS.

The Attenuated total reflect Fourier Transform infrared spectroscopy (ATR FT-IR) spectra of HAs and HAs derivatives (MEHA and MAHA) are presented in figure S2. For MEHA (Fig. S2a), it is possible to asses that the band extending between 950 and 1200 cm^-1^ corresponds to the C-O stretching vibrations (ν_C-OH_), the intense group of bands that extends from 1500 to 1700 cm^-1^ represents the superposition of amide I and II and of various carbonyl and carboxyl ν_C=O_ bands. In particular, the peak at 1719 cm^−1^ is featured by ν_C=O_ of the methacrylic moiety. Similarly, FT-IR spectrum of MAHA (Fig. S2b) shows characteristic peaks at 1576 cm^-1^ related to the o the α,β -unsaturated ester/acid ( -CO-C=C-CO- ) of the modified HAs. With the increase of the DS, an intensity increase of the characteristic peaks both for MEHA and MAHA was observed.


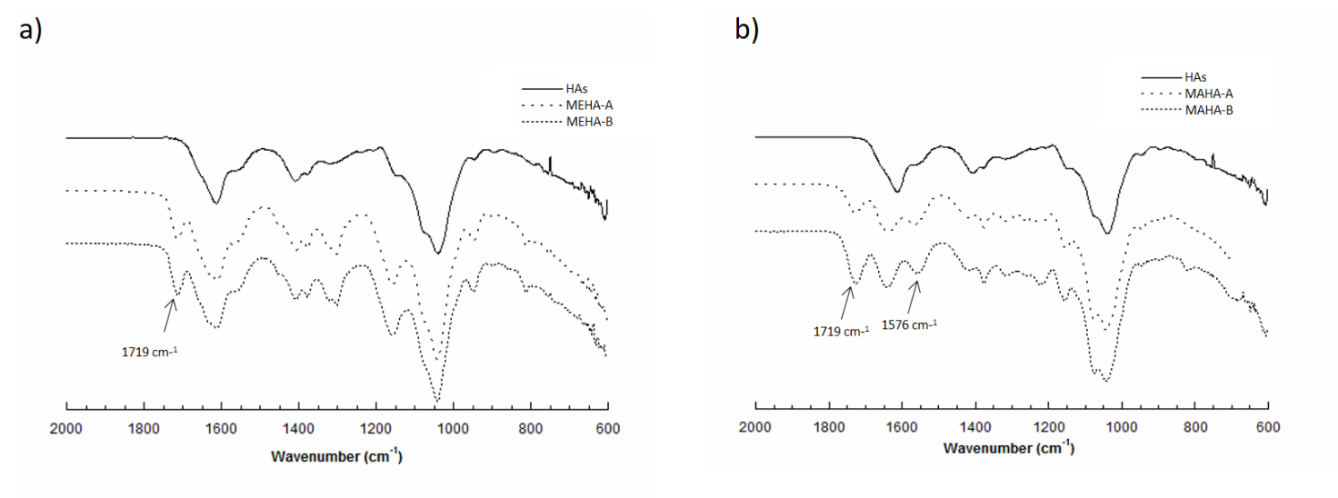


Figure S2. The ATR FT-IR spectra of a) MEHA and b) MAHA with different DS.

**Morphological investigation**

In order to assess the presence of CaP, elemental distribution analysis was carried out for each group. As an example, the SEM–EDS P- and Ca-mapping photographs of MEHA-B/CaP25 and MAHA-B/CaP25, are presented in figures S3a and b. Calcium was labelled blue while phosphorus was labelled green. It can be seen that particles and plate-like CaP are homogenously distributed inside the polymer matrix.


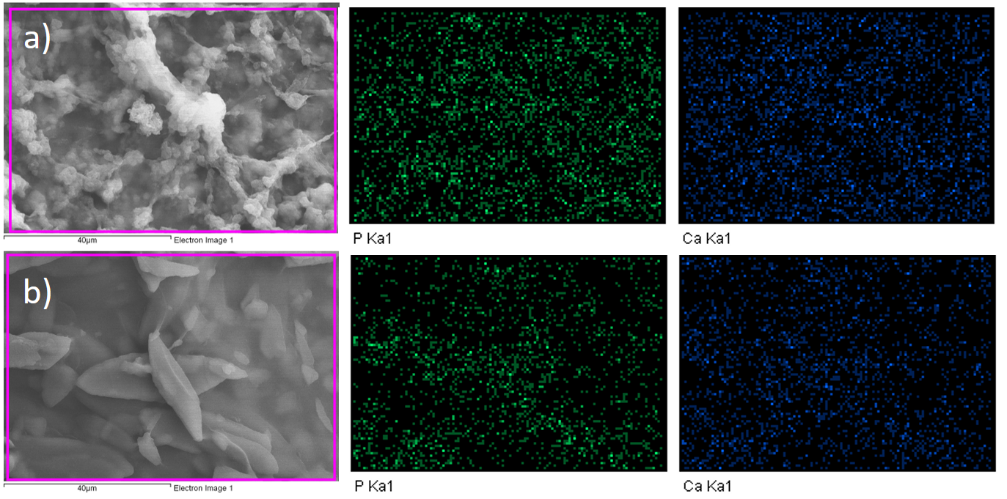


Figure S3. SEM–EDS P- and Ca-mapping photographs of a) MEHA-B/CaP25; b) MAHA-B/CaP25

Results from Toluidine blue and Alizarin Red staining confirmed the presence of CaP fillers (red dots in the figures) in the polymer matrix (purple area in the Fig. S4a-b). The fillers were well distributed in the HAs matrix, without a significant difference between top, middle and bottom side of the composite hydrogel. As example, in figures S4 the representative images of MEHA and MAHA-based composites are reported.


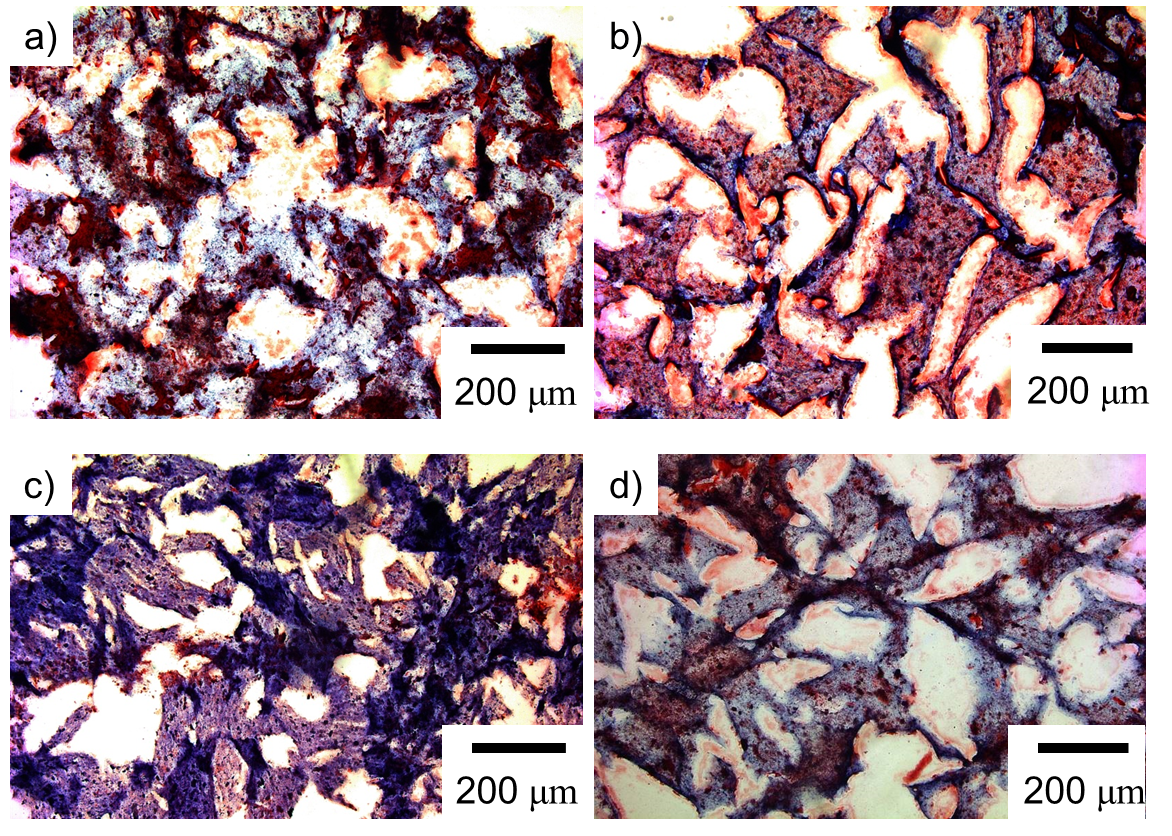


Figure S4. Representative image of Toluidine Blue and Alizarin Red staining performed on MEHA (a, b) and MAHA (c, d) based composites: a), c) CaP25, b), d) CaP50.

**Reference**

1. Vasi AM, Popa MI, Butnaru M et al. Chemical functionalization of hyaluronic acid for drug delivery applications. *Mater Sci Eng C* 2014;38:177-185.
